# The effects of Ca^2+^ buffers on cytosolic Ca^2+^ signalling

**DOI:** 10.1113/JP273852

**Published:** 2017-02-01

**Authors:** Ole H. Petersen

**Affiliations:** ^1^ School of Biosciences, Sir Martin Evans BuildingCardiff UniversityCardiffUK; ^2^ Systems Immunity Research InstituteCardiff UniversityCardiffUK

**Keywords:** calcium buffer, calcium dynamics, calcium signalling

Ca^2+^ is one of the most important and universal intracellular signalling agents, controlling a multitude of vitally important cellular processes (Petersen & Verkhratsky 2016). The regulating cytosolic Ca^2+^ signals vary enormously with regard to timing, localization and spatial extent, depending on the specific cell type and the function to be controlled (Petersen & Verkhratsky, 2016). Because of the many different types of Ca^2+^ sensors (Ca^2+^‐binding proteins) found at different locations inside cells, there is often a requirement for Ca^2+^ signals to be strictly localized. A well‐known example of this is the control of neurotransmitter release by Ca^2+^ entry through voltage‐gated Ca^2+^ channels, generating short‐lived local nano‐domains of high Ca^2+^ concentration ([Ca^2+^]_i_) (Meinrenken *et al*. 2003). A more recent, and very interesting, example is the physiological activation of two Ca^2+^‐dependent transcription isoforms, NFAT1 and NFAT4. This occurs through two coincident, but spatially segregated, intracellular Ca^2+^ signals, namely in the immediate vicinity of Ca^2+^ release‐activated Ca^2+^ channels in the plasma membrane and close to inositol trisphosphate receptors in the inner nuclear membrane (Kar *et al*. 2016).

Different spatio‐temporal Ca^2+^ signal patterns can occur in the same cell depending on the type, intensity and duration of stimulation (Petersen 1992). In pancreatic acinar cells, both local Ca^2+^ spiking in the region of secretory control and global [Ca^2+^]_i_ elevations can be generated, which is important because Ca^2+^ signals control not only secretion but also many other functions, including growth (cell division) (Petersen, 1992). In nerve terminals, functions other than neurotransmitter secretion may depend on the global [Ca^2+^]_i_. There has therefore, for a long time, been much interest in measuring the volume‐averaged [Ca^2+^]_i_ (Meinrenken *et al*. 2003), which can also be particularly helpful for a precise quantitative evaluation of Ca^2+^ handling.

The nature and concentration of intracellular Ca^2+^ buffers play an important role in determining the timing and spreading of Ca^2+^ released into the cytosol by opening of Ca^2+^ channels either in the plasma membrane or in organelle membranes. An early example of experiments showing this phenomenon was the demonstration, in whole‐cell patch clamp current recording studies on pancreatic acinar cells, that intracellular addition of a highly mobile low‐affinity Ca^2+^ buffer, for example citrate, transformed ACh‐evoked local and short‐lasting Ca^2+^ spikes into global and much more prolonged Ca^2+^ transients (Petersen, 1992). The timing and spatial extension of physiological Ca^2+^ signals therefore depends not only on the strength and type of stimulation but also on the affinities, mobilities and concentrations of the various intracellular Ca^2+^ buffers, which can vary significantly between different cell types. There is also a practical issue relating to the ability of Ca^2+^ buffers to influence intracellular Ca^2+^ signals. Because all Ca^2+^‐sensitive fluorescent probes are Ca^2+^ buffers, they can distort the [Ca^2+^]_i_ signals they are designed to monitor.

It has been challenging to obtain reliable estimates for the parameters that define the dynamics of physiological [Ca^2+^]_i_ changes. In this issue of *The Journal of Physiology*, Erwin Neher and his colleagues (Lin *et al*. 2017) now describe the currently most precise quantitative approach to solving this problem by once more taking advantage of the calyx of Held, a giant mammalian glutamatergic nerve terminal which – following the pioneering work of Ian Forsythe (Forsythe, 1994) – has been extensively studied by several groups (Meinrenken *et al*. 2003). Lin *et al*. (2017) describe the dynamic changes of global [Ca^2+^]_i_ during single and repetitive voltage‐clamp depolarizations and provide quantitative data on Ca^2+^ inflow, Ca^2+^ buffering and Ca^2+^ clearance.

Using low concentrations of the low‐affinity Ca^2+^ indicator Fura‐6F, in order not to overwhelm the endogenous Ca^2+^ buffers, Lin *et al*. (2017) studied the voltage‐clamped nerve terminals with patch pipettes containing solutions with minimal Ca^2+^ buffer concentrations. This allowed them to determine the Ca^2+^ binding properties of the endogenous fixed buffers and also the Ca^2+^ clearance mechanism. With regard to the latter, a comparison was made between the results obtained with Cs^+^‐ or K^+^‐based pipette solutions. The data from these experiments confirmed the importance of K^+^‐dependent Na^+^–Ca^2+^ exchange for Ca^2+^ extrusion (Schnetkamp, 2004). In other experiments, Lin *et al*. (2017) used pipette solutions with 500 μm of the widely used Ca^2+^ chelator EGTA, determining its Ca^2+^ binding characteristics under realistic intracellular conditions. It turned out that the Ca^2+^ dissociation constant of EGTA is more than 3 times higher than the value previously obtained *in vitro*. This result is of great practical importance as EGTA has been, and no doubt will continue to be, a useful tool as a slow Ca^2+^ chelator buffering global rather than local [Ca^2+^]_i_.

Overall, the major importance of the work reported by Lin *et al*. (2017) is that, based on very sensitive experimental protocols, they have been able to generate a consistent set of parameters for modelling [Ca^2+^]_i_ transients in a mammalian presynaptic nerve terminal. Estimates of some of the parameters determining [Ca^2+^]_i_ dynamics have been reported previously, but they were based on experiments carried out under a variety of different conditions, whereas the new study by Lin *et al*. (2017) has resulted in a comprehensive set of parameters valid for recording conditions generally used for studies of the calyx of Held. The results of the work of Lin *et al*. (2017) that ‘one set of parameters accurately describes [Ca^2+^]_i_ measurements covering a wide range of amplitudes and obtained using quite different stimulation protocols and ionic conditions’ is remarkable and promises that this set will turn out to be of real help as a firm quantitative basis for further studies in this field.

## Additional information

### Competing interests

No competing interests declared.

### Funding

The author is a Medical Research Council Professor (G19/22/2) and is supported by a Medical Research Council Programme grant (MR/J002771/1).
